# *rac*-5-Bromo-*N*-benzyl­isatincreatinine ethanol monosolvate

**DOI:** 10.1107/S160053681300038X

**Published:** 2013-01-26

**Authors:** Narsimha Reddy Penthala, Peter A. Crooks

**Affiliations:** a Department of Pharmaceutical Sciences, College of Pharmacy, University of Arkansas for Medical Sciences, Little Rock, AR 72205, USA

## Abstract

In the title compound [systematic name: *rac*-1-benzyl-5-bromo-3-hy­droxy-3-(2-imino-3-methyl-5-oxoimidazolidin-4-yl)-2,3-dihydro-1*H*-indol-2-one ethanol monosolvate], C_19_H_17_BrN_4_O_3_·C_2_H_5_OH, which crystallized as a racemate (*RR* and *SS*), the isatin ring is almost planar, with an r.m.s. deviations from the mean plane of 0.0276 (14) Å. The phenyl ring of the benzyl group makes a dihedral angle with the mean plane of the isatin ring of 87.40 (5)° and the dihedral angle between the imidazole and isatin rings is 58.56 (7)°. In the crystal, mol­ecules are linked into two-dimensional pleated-sheet networks in the *ac* plane formed by O—H⋯O, N—H⋯O and O—H⋯N hydrogen bonds; within these sheets there are *R*_4_^4^(10) rings that involve three mol­ecules of the title compound and a single ethanol solvent mol­ecule. In addition, there are π–π inter­actions between inversion-related benzyl groups, with an inter­planar spacing of 3.444 (3) Å.

## Related literature

Background information on the biological importance of isatins has been given by Pandeya *et al.* (2005 ▶), and by Vine *et al.* (2007 ▶). For similar structures, see: Tang *et al.* (2009 ▶); Penthala *et al.* (2009*a* ▶,*b* ▶).
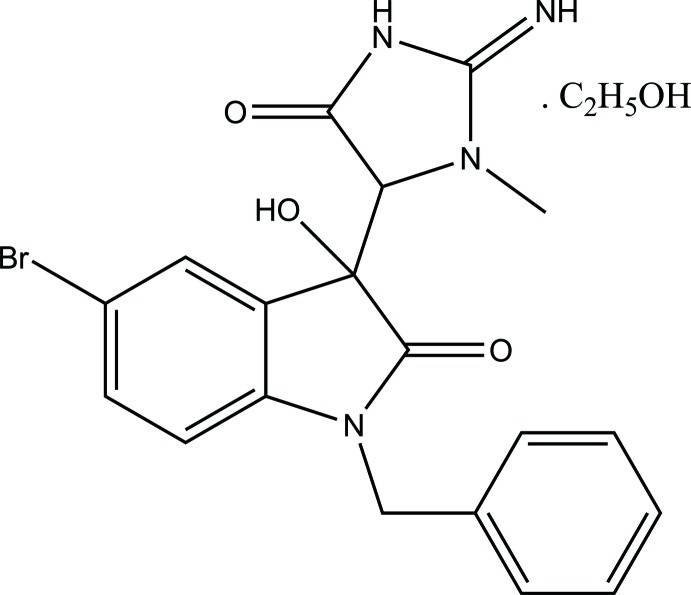


## Experimental

### 

#### Crystal data

C_19_H_17_BrN_4_O_3_·C_2_H_6_O
*M* *_r_* = 475.34
Monoclinic, 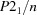

*a* = 7.8384 (16) Å
*b* = 24.553 (5) Å
*c* = 10.936 (2) Å
β = 99.54 (3)°
*V* = 2075.6 (7) Å^3^
*Z* = 4
Mo *K*α radiation
μ = 2.02 mm^−1^
*T* = 90 K
0.20 × 0.15 × 0.10 mm

#### Data collection

Nonius KappaCCD diffractometer
Absorption correction: multi-scan [*SCALEPACK* (Otwinowski & Minor, 1997 ▶) and *XABS2* (Parkin *et al.*, 1995 ▶)] *T*_min_ = 0.689, *T*_max_ = 0.824
41507 measured reflections
4752 independent reflections
4147 reflections with *I* > 2σ(*I*)
*R*_int_ = 0.038

#### Refinement

*R*[*F* ^2^ > 2σ(*F* ^2^)] = 0.029
*wR*(*F* ^2^) = 0.074
*S* = 1.05
4752 reflections
282 parameters
3 restraints
H atoms treated by a mixture of independent and constrained refinement
Δρ_max_ = 1.19 e Å^−3^
Δρ_min_ = −0.45 e Å^−3^

### 

Data collection: *COLLECT* (Nonius, 1998 ▶); cell refinement: *SCALEPACK* (Otwinowski & Minor, 1997 ▶); data reduction: *DENZO-SMN* (Otwinowski & Minor, 1997 ▶); program(s) used to solve structure: *SHELXS97* (Sheldrick, 2008 ▶); program(s) used to refine structure: *SHELXL97* (Sheldrick, 2008 ▶); molecular graphics: *XP* in *SHELXTL* (Sheldrick, 2008 ▶); software used to prepare material for publication: *SHELXL97* and local procedures.

## Supplementary Material

Crystal structure: contains datablock(s) global, I. DOI: 10.1107/S160053681300038X/hg5279sup1.cif

Structure factors: contains datablock(s) I. DOI: 10.1107/S160053681300038X/hg5279Isup2.hkl

Supplementary material file. DOI: 10.1107/S160053681300038X/hg5279Isup3.cml

Additional supplementary materials:  crystallographic information; 3D view; checkCIF report

## Figures and Tables

**Table 1:** Hydrogen-bond geometry (Å, °)

*D*—H⋯*A*	*D*—H	H⋯*A*	*D*⋯*A*	*D*—H⋯*A*
O9—H9⋯O1*S* ^i^	0.84	1.87	2.694 (2)	169
N13—H13*A*⋯O11^ii^	0.81 (2)	2.00 (2)	2.811 (2)	171 (2)
N13—H13*B*⋯O9^iii^	0.82 (2)	2.36 (2)	2.933 (2)	128 (2)
N13—H13*B*⋯O1^iii^	0.82 (2)	2.46 (2)	3.158 (2)	144 (2)
O1*S*—H1*S*⋯N12	0.84	1.91	2.745 (2)	174

Symmetry codes: (i) 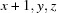; (ii) 
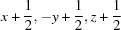
; (iii) 
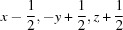
.

**Fig. 1.**
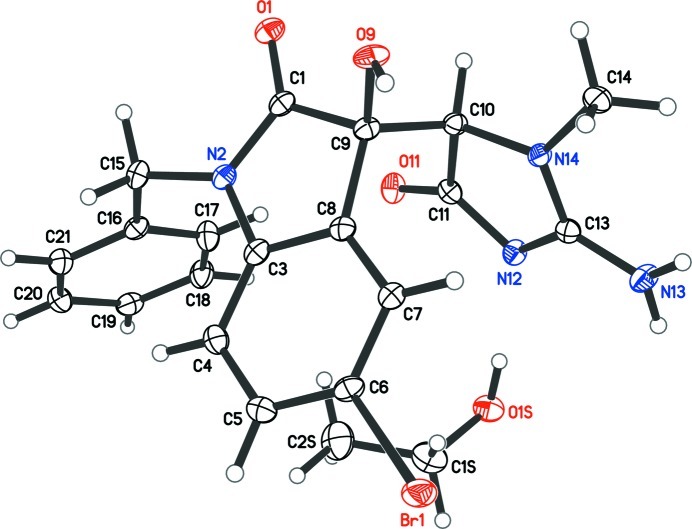
A view of displacement ellipsoids drawn at the 50% probability level.

**Fig. 2.**
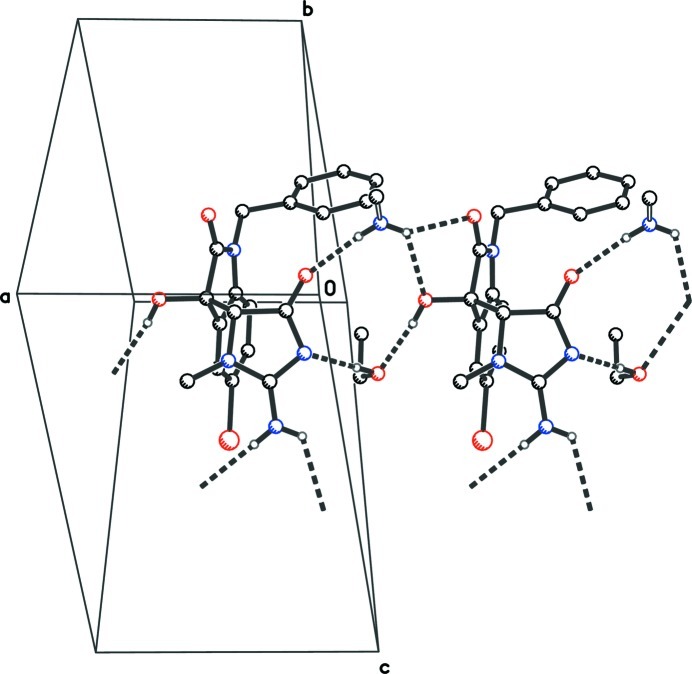
Hydrogen bonding in the crystal structure of the title compound. Dashed lines represent hydrogen bonds, while the open bonds indicate continuation of the structure, approximately in the (0 1 - 1) direction.

## supplementary crystallographic information

### Comment

In view of the biological importance of isatins (Pandeya *et al.*, 2005; Vine *et al.*, 2007) we have synthesized a series of novel compounds containing isatin and creatinine moieties to screen for their anticancer activity. The title compound was prepared by the aldol condensation of 5-bromo-*N*-benzylindol-2,3-dione (5-bromo-*N*-benzylisatin) with 2-amino-1-methyl-1*H*-imidazol-4(5*H*)-one (creatinine) in the presence of sodium acetate in acetic acid. Earlier, we reported on the crystal structure of isatin creatinine analogs containing *N*-methyl and *N*-phenyl substitituents (Penthala *et al.*, 2009*a*,*b*). To obtain detailed information on the structural conformations of the molecules for analysis of structure-activity relationships (SAR), we determined the X-ray crystal structure of the title compound. The compound crystallized as a racemate (*RR* and *SS*). The molecular structure of title compound is shown in Fig. 1. The isatin ring is almost planar with r.m.s deviation from the mean plane of 0.0276 (14) Å, with bond distances and angles comparable to those reported for other isatin derivatives (Tang *et al.*, 2009). The benzene ring of the benzyl group makes a dihedral angle with the mean plane of the isatin ring of 87.40 (5)°. In the title compound the molecules are linked into 2-D pleated-sheet networks in the *ac* plane by O—H···O, N—H···O and O—H···N hydrogen bonds. Within these sheets there are *R*^4^_4_(10) rings that involve three molecules of the title compound and a single ethanol solvent molecule.

### Experimental

The title compound was prepared according to a previously reported procedure (Penthala *et al.*, 2009*a*,*b*). Recrystallization from ethanol afforded the title compound as a pale-yellow crystalline product that was suitable for X-ray analysis. Spectroscopic data for *rac*-5-bromo-*N*-benzylisatin creatinine: ^1^H NMR(DMSO-*d_6_*): δ 3.2 0 (s, 3H, CH_3_), 4.22 (s, 1H, CH), 4.74–4.92 (ABq, 2H, CH_2_), 6.62–6.64 (d, *J*=8.1 Hz, 1H, C_7_H), 6.74 (s, 1H, OH), 7.19–7.20 (d, *J*=1.5 Hz, 1H,-C_4_H), 7.25–7.46 (m, 6H, C_5_H, C_6_H, Ar—H), 7.82 (bs, 2H, NH_2_); ^13^C NMR (DMSO-*d_6_*): δ 32.71, 42.76, 69.61, 75.87, 110.43, 123.55, 125.67, 126.89, 127.05 (2 C), 128.21 (2 C), 129.01, 129.17, 135.39, 141.91, 172.10 (C=N), 173.98 (isatin C=O), 181.93 (creatinine C=O).

### Refinement

All H atoms were found in difference Fourier maps. All except those attached to nitrogen were subsequently placed at idealized positions with constrained distances of 0.98 Å (RCH_3_), 0.99 Å (*R*_2_CH_2_), 1.00 Å (*R*_3_CH), 0.95 Å (C_Ar_H) and 0.84 Å (O—H). N-bound H atoms were refined with 1,2 and 1,3 distance restraints (*DFIX* in *SHELXL97*). *U*_iso_(H) values were set to either 1.2*U*_eq_ or 1.5*U*_eq_ (RCH_3_, OH) of the attached atom.

The largest difference map peak (~1.2 e Å^3^) is about 1.87 Å away from C7, and so could conceivably represent a very minor impurity in which the Br atom is attached to C7 rather than C5. Such a disorder model was made, and it refined in a stable manner with a refined occupancy of <2%. Although the difference map was flatter and it had very good 'quality' statistics, there seemed little point in keeping this disorder model because the occupancy was so low.

### Crystal data

**Table:**

C_19_H_17_BrN_4_O_3_·C_2_H_6_O	*F*(000) = 976
*M**_r_* = 475.34	*D*_x_ = 1.521 Mg m^−^^3^
Monoclinic, *P*2_1_/*n*	Mo *K*α radiation, λ = 0.71073 Å
Hall symbol: -P 2yn	Cell parameters from 51652 reflections
*a* = 7.8384 (16) Å	θ = 1.0–27.5°
*b* = 24.553 (5) Å	µ = 2.02 mm^−^^1^
*c* = 10.936 (2) Å	*T* = 90 K
β = 99.54 (3)°	Block, colourless
*V* = 2075.6 (7) Å^3^	0.20 × 0.15 × 0.10 mm
*Z* = 4	

### Data collection

**Table:**

Nonius KappaCCD diffractometer	4752 independent reflections
Radiation source: fine-focus sealed tube	4147 reflections with *I* > 2σ(*I*)
Graphite monochromator	*R*_int_ = 0.038
Detector resolution: 9.1 pixels mm^-1^	θ_max_ = 27.5°, θ_min_ = 1.7°
ω scans at fixed χ = 55°	*h* = −10→10
Absorption correction: multi-scan [*SCALEPACK* (Otwinowski & Minor, 1997) and *XABS2* (Parkin *et al.*, 1995)]	*k* = −31→31
*T*_min_ = 0.689, *T*_max_ = 0.824	*l* = −14→14
41507 measured reflections	

### Refinement

**Table:**

Refinement on *F*^2^	Primary atom site location: structure-invariant direct methods
Least-squares matrix: full	Secondary atom site location: difference Fourier map
*R*[*F*^2^ > 2σ(*F*^2^)] = 0.029	Hydrogen site location: inferred from neighbouring sites
*wR*(*F*^2^) = 0.074	H atoms treated by a mixture of independent and constrained refinement
*S* = 1.05	*w* = 1/[σ^2^(*F*_o_^2^) + (0.0318*P*)^2^ + 2.2794*P*] where *P* = (*F*_o_^2^ + 2*F*_c_^2^)/3
4752 reflections	(Δ/σ)_max_ = 0.002
282 parameters	Δρ_max_ = 1.19 e Å^−^^3^
3 restraints	Δρ_min_ = −0.45 e Å^−^^3^

### Special details

**Table:**

Geometry. All e.s.d.'s (except the e.s.d. in the dihedral angle between two l.s. planes) are estimated using the full covariance matrix. The cell e.s.d.'s are taken into account individually in the estimation of e.s.d.'s in distances, angles and torsion angles; correlations between e.s.d.'s in cell parameters are only used when they are defined by crystal symmetry. An approximate (isotropic) treatment of cell e.s.d.'s is used for estimating e.s.d.'s involving l.s. planes.
Refinement. Refinement of *F*^2^ against all reflections. The weighted *R*-value *wR* and goodness of fit *S* are based on *F*^2^. Conventional *R*-values *R* are based on *F*, with *F* set to zero for negative *F*^2^. The threshold expression of *F*^2^ > 2σ(*F*^2^) is used only for calculating *R*-factors(gt) *etc*. and is not relevant to the choice of reflections for refinement. *R*-values based on *F*^2^ are statistically about twice as large as those based on *F*, and *R*-values based on ALL data will be even larger.

### Fractional atomic coordinates and isotropic or equivalent isotropic displacement parameters (Å^2^)

**Table:**

	*x*	*y*	*z*	*U*_iso_*/*U*_eq_	
Br1	0.49690 (3)	0.374439 (8)	0.816959 (17)	0.02004 (7)	
O1	0.41024 (17)	0.35297 (6)	0.10785 (12)	0.0171 (3)	
C1	0.4044 (2)	0.36264 (7)	0.21623 (17)	0.0130 (3)	
N2	0.35261 (19)	0.41049 (6)	0.26345 (14)	0.0131 (3)	
C3	0.3743 (2)	0.40838 (7)	0.39354 (17)	0.0127 (3)	
C4	0.3378 (2)	0.44865 (8)	0.47375 (18)	0.0152 (4)	
H4	0.2896	0.4825	0.4435	0.018*	
C5	0.3745 (2)	0.43786 (8)	0.60104 (17)	0.0160 (4)	
H5	0.3503	0.4646	0.6587	0.019*	
C6	0.4460 (2)	0.38823 (8)	0.64338 (16)	0.0140 (4)	
C7	0.4822 (2)	0.34757 (7)	0.56274 (16)	0.0129 (3)	
H7	0.5311	0.3138	0.5931	0.015*	
C8	0.4445 (2)	0.35808 (7)	0.43654 (17)	0.0121 (3)	
O9	0.64140 (16)	0.31074 (6)	0.31688 (12)	0.0163 (3)	
H9	0.7024	0.3118	0.3877	0.024*	
C9	0.4680 (2)	0.32370 (7)	0.32616 (16)	0.0124 (3)	
C10	0.3592 (2)	0.27084 (7)	0.31485 (16)	0.0123 (3)	
H10	0.3696	0.2511	0.2364	0.015*	
O11	0.07782 (17)	0.31156 (5)	0.24552 (12)	0.0169 (3)	
C11	0.1692 (2)	0.28271 (7)	0.32180 (16)	0.0127 (3)	
N12	0.12428 (19)	0.25919 (6)	0.42372 (14)	0.0137 (3)	
N13	0.2659 (2)	0.20205 (7)	0.57992 (15)	0.0166 (3)	
H13A	0.356 (3)	0.1946 (9)	0.625 (2)	0.020*	
H13B	0.175 (3)	0.2005 (10)	0.608 (2)	0.020*	
C13	0.2659 (2)	0.23119 (7)	0.47904 (16)	0.0128 (3)	
N14	0.40463 (19)	0.23525 (6)	0.42122 (14)	0.0121 (3)	
C14	0.5592 (2)	0.20123 (8)	0.44579 (18)	0.0177 (4)	
H14A	0.6453	0.2187	0.5088	0.026*	
H14B	0.6073	0.1967	0.3692	0.026*	
H14C	0.5288	0.1655	0.4758	0.026*	
C15	0.3028 (2)	0.45903 (8)	0.18962 (17)	0.0156 (4)	
H15A	0.3328	0.4536	0.1060	0.019*	
H15B	0.3718	0.4902	0.2282	0.019*	
C16	0.1127 (2)	0.47366 (8)	0.17564 (16)	0.0146 (4)	
C17	−0.0132 (3)	0.43353 (8)	0.17080 (19)	0.0197 (4)	
H17	0.0203	0.3965	0.1834	0.024*	
C18	−0.1880 (3)	0.44717 (8)	0.14763 (19)	0.0204 (4)	
H18	−0.2733	0.4195	0.1438	0.024*	
C19	−0.2373 (3)	0.50119 (8)	0.13010 (18)	0.0187 (4)	
H19	−0.3564	0.5106	0.1133	0.022*	
C20	−0.1119 (3)	0.54152 (8)	0.13722 (19)	0.0198 (4)	
H20	−0.1454	0.5786	0.1266	0.024*	
C21	0.0624 (3)	0.52780 (8)	0.15986 (18)	0.0181 (4)	
H21	0.1476	0.5556	0.1646	0.022*	
O1S	−0.13483 (17)	0.30418 (6)	0.53172 (13)	0.0191 (3)	
H1S	−0.0600	0.2885	0.4975	0.029*	
C1S	−0.0616 (3)	0.35257 (9)	0.5930 (2)	0.0249 (4)	
H1S1	0.0641	0.3470	0.6199	0.030*	
H1S2	−0.1143	0.3588	0.6680	0.030*	
C2S	−0.0892 (3)	0.40229 (9)	0.5114 (2)	0.0300 (5)	
H2S1	−0.0366	0.3965	0.4372	0.045*	
H2S2	−0.0355	0.4339	0.5570	0.045*	
H2S3	−0.2135	0.4088	0.4869	0.045*	

### Atomic displacement parameters (Å^2^)

**Table:**

	*U*^11^	*U*^22^	*U*^33^	*U*^12^	*U*^13^	*U*^23^
Br1	0.02698 (11)	0.02127 (11)	0.01143 (10)	0.00486 (8)	0.00183 (7)	−0.00050 (7)
O1	0.0183 (7)	0.0219 (7)	0.0113 (6)	−0.0017 (6)	0.0030 (5)	0.0000 (5)
C1	0.0084 (8)	0.0169 (9)	0.0139 (9)	−0.0023 (6)	0.0026 (6)	0.0011 (7)
N2	0.0139 (7)	0.0146 (7)	0.0108 (7)	−0.0001 (6)	0.0016 (6)	0.0019 (6)
C3	0.0094 (8)	0.0160 (9)	0.0125 (8)	−0.0025 (7)	0.0010 (6)	0.0011 (7)
C4	0.0140 (8)	0.0131 (8)	0.0180 (9)	0.0005 (7)	0.0014 (7)	0.0005 (7)
C5	0.0157 (9)	0.0167 (9)	0.0157 (9)	−0.0001 (7)	0.0029 (7)	−0.0033 (7)
C6	0.0130 (8)	0.0191 (9)	0.0094 (8)	−0.0015 (7)	0.0005 (7)	−0.0006 (7)
C7	0.0105 (8)	0.0151 (9)	0.0129 (8)	0.0000 (7)	0.0016 (7)	0.0012 (7)
C8	0.0081 (8)	0.0144 (8)	0.0140 (9)	−0.0019 (6)	0.0028 (6)	−0.0006 (7)
O9	0.0104 (6)	0.0260 (7)	0.0129 (6)	0.0032 (5)	0.0033 (5)	−0.0005 (5)
C9	0.0103 (8)	0.0157 (9)	0.0113 (8)	0.0014 (7)	0.0019 (6)	0.0010 (7)
C10	0.0129 (8)	0.0137 (8)	0.0102 (8)	0.0018 (7)	0.0016 (6)	0.0004 (7)
O11	0.0148 (6)	0.0168 (7)	0.0174 (7)	−0.0004 (5)	−0.0025 (5)	0.0033 (5)
C11	0.0111 (8)	0.0121 (8)	0.0138 (9)	−0.0012 (6)	−0.0006 (7)	−0.0023 (7)
N12	0.0112 (7)	0.0151 (7)	0.0144 (7)	0.0011 (6)	0.0013 (6)	0.0023 (6)
N13	0.0123 (8)	0.0232 (9)	0.0146 (8)	0.0022 (7)	0.0033 (6)	0.0049 (6)
C13	0.0123 (8)	0.0130 (8)	0.0128 (8)	−0.0010 (7)	0.0014 (7)	−0.0015 (7)
N14	0.0117 (7)	0.0134 (7)	0.0116 (7)	0.0031 (6)	0.0031 (6)	0.0025 (6)
C14	0.0159 (9)	0.0185 (9)	0.0197 (10)	0.0076 (7)	0.0063 (7)	0.0034 (7)
C15	0.0160 (9)	0.0150 (9)	0.0155 (9)	−0.0009 (7)	0.0019 (7)	0.0044 (7)
C16	0.0168 (9)	0.0173 (9)	0.0095 (8)	0.0000 (7)	0.0017 (7)	0.0019 (7)
C17	0.0192 (10)	0.0152 (9)	0.0243 (10)	0.0004 (7)	0.0026 (8)	0.0040 (8)
C18	0.0175 (9)	0.0189 (10)	0.0254 (10)	−0.0036 (8)	0.0051 (8)	0.0021 (8)
C19	0.0179 (9)	0.0234 (10)	0.0152 (9)	0.0041 (8)	0.0044 (7)	0.0023 (8)
C20	0.0232 (10)	0.0153 (9)	0.0214 (10)	0.0034 (8)	0.0055 (8)	0.0009 (7)
C21	0.0201 (9)	0.0164 (9)	0.0182 (9)	−0.0028 (8)	0.0044 (7)	−0.0006 (7)
O1S	0.0127 (6)	0.0229 (7)	0.0217 (7)	0.0024 (5)	0.0033 (5)	−0.0021 (6)
C1S	0.0218 (10)	0.0275 (11)	0.0240 (11)	0.0005 (9)	−0.0002 (8)	−0.0052 (9)
C2S	0.0239 (11)	0.0286 (12)	0.0376 (13)	−0.0076 (9)	0.0049 (10)	0.0006 (10)

### Geometric parameters (Å, º)

**Table:**

Br1—C6	1.9042 (18)	C13—N14	1.349 (2)
O1—C1	1.217 (2)	N14—C14	1.460 (2)
C1—N2	1.372 (2)	C14—H14A	0.9800
C1—C9	1.552 (2)	C14—H14B	0.9800
N2—C3	1.406 (2)	C14—H14C	0.9800
N2—C15	1.456 (2)	C15—C16	1.516 (3)
C3—C4	1.383 (3)	C15—H15A	0.9900
C3—C8	1.401 (3)	C15—H15B	0.9900
C4—C5	1.399 (3)	C16—C17	1.389 (3)
C4—H4	0.9500	C16—C21	1.389 (3)
C5—C6	1.388 (3)	C17—C18	1.392 (3)
C5—H5	0.9500	C17—H17	0.9500
C6—C7	1.392 (3)	C18—C19	1.386 (3)
C7—C8	1.387 (3)	C18—H18	0.9500
C7—H7	0.9500	C19—C20	1.388 (3)
C8—C9	1.509 (2)	C19—H19	0.9500
O9—C9	1.416 (2)	C20—C21	1.389 (3)
O9—H9	0.8400	C20—H20	0.9500
C9—C10	1.546 (2)	C21—H21	0.9500
C10—N14	1.451 (2)	O1S—C1S	1.436 (3)
C10—C11	1.532 (2)	O1S—H1S	0.8400
C10—H10	1.0000	C1S—C2S	1.506 (3)
O11—C11	1.230 (2)	C1S—H1S1	0.9900
C11—N12	1.353 (2)	C1S—H1S2	0.9900
N12—C13	1.359 (2)	C2S—H2S1	0.9800
N13—C13	1.315 (2)	C2S—H2S2	0.9800
N13—H13A	0.81 (2)	C2S—H2S3	0.9800
N13—H13B	0.82 (2)		
			
O1—C1—N2	126.51 (17)	C13—N14—C10	108.09 (14)
O1—C1—C9	125.11 (17)	C13—N14—C14	125.49 (15)
N2—C1—C9	108.24 (15)	C10—N14—C14	125.31 (15)
C1—N2—C3	110.84 (15)	N14—C14—H14A	109.5
C1—N2—C15	124.14 (16)	N14—C14—H14B	109.5
C3—N2—C15	124.60 (15)	H14A—C14—H14B	109.5
C4—C3—C8	121.89 (17)	N14—C14—H14C	109.5
C4—C3—N2	127.78 (17)	H14A—C14—H14C	109.5
C8—C3—N2	110.31 (16)	H14B—C14—H14C	109.5
C3—C4—C5	117.77 (17)	N2—C15—C16	114.75 (15)
C3—C4—H4	121.1	N2—C15—H15A	108.6
C5—C4—H4	121.1	C16—C15—H15A	108.6
C6—C5—C4	120.19 (17)	N2—C15—H15B	108.6
C6—C5—H5	119.9	C16—C15—H15B	108.6
C4—C5—H5	119.9	H15A—C15—H15B	107.6
C5—C6—C7	122.12 (17)	C17—C16—C21	119.26 (18)
C5—C6—Br1	119.66 (14)	C17—C16—C15	121.06 (17)
C7—C6—Br1	118.22 (14)	C21—C16—C15	119.56 (17)
C8—C7—C6	117.69 (17)	C16—C17—C18	120.50 (18)
C8—C7—H7	121.2	C16—C17—H17	119.7
C6—C7—H7	121.2	C18—C17—H17	119.7
C7—C8—C3	120.34 (17)	C19—C18—C17	119.93 (18)
C7—C8—C9	131.13 (17)	C19—C18—H18	120.0
C3—C8—C9	108.53 (15)	C17—C18—H18	120.0
C9—O9—H9	109.5	C18—C19—C20	119.74 (18)
O9—C9—C8	115.38 (15)	C18—C19—H19	120.1
O9—C9—C10	109.18 (14)	C20—C19—H19	120.1
C8—C9—C10	113.26 (14)	C19—C20—C21	120.23 (18)
O9—C9—C1	105.76 (14)	C19—C20—H20	119.9
C8—C9—C1	102.06 (14)	C21—C20—H20	119.9
C10—C9—C1	110.69 (14)	C20—C21—C16	120.32 (18)
N14—C10—C11	100.72 (14)	C20—C21—H21	119.8
N14—C10—C9	112.50 (14)	C16—C21—H21	119.8
C11—C10—C9	111.34 (14)	C1S—O1S—H1S	109.5
N14—C10—H10	110.6	O1S—C1S—C2S	112.79 (18)
C11—C10—H10	110.6	O1S—C1S—H1S1	109.0
C9—C10—H10	110.6	C2S—C1S—H1S1	109.0
O11—C11—N12	127.07 (17)	O1S—C1S—H1S2	109.0
O11—C11—C10	122.53 (16)	C2S—C1S—H1S2	109.0
N12—C11—C10	110.35 (15)	H1S1—C1S—H1S2	107.8
C11—N12—C13	106.12 (15)	C1S—C2S—H2S1	109.5
C13—N13—H13A	121.0 (16)	C1S—C2S—H2S2	109.5
C13—N13—H13B	117.3 (16)	H2S1—C2S—H2S2	109.5
H13A—N13—H13B	118 (2)	C1S—C2S—H2S3	109.5
N13—C13—N14	123.05 (17)	H2S1—C2S—H2S3	109.5
N13—C13—N12	122.36 (16)	H2S2—C2S—H2S3	109.5
N14—C13—N12	114.59 (16)		
			
O1—C1—N2—C3	−175.80 (17)	C8—C9—C10—N14	−61.17 (19)
C9—C1—N2—C3	0.11 (19)	C1—C9—C10—N14	−175.10 (14)
O1—C1—N2—C15	−2.9 (3)	O9—C9—C10—C11	−178.91 (14)
C9—C1—N2—C15	172.95 (15)	C8—C9—C10—C11	51.04 (19)
C1—N2—C3—C4	179.53 (18)	C1—C9—C10—C11	−62.89 (18)
C15—N2—C3—C4	6.7 (3)	N14—C10—C11—O11	−178.92 (16)
C1—N2—C3—C8	0.7 (2)	C9—C10—C11—O11	61.6 (2)
C15—N2—C3—C8	−172.11 (16)	N14—C10—C11—N12	3.60 (19)
C8—C3—C4—C5	0.4 (3)	C9—C10—C11—N12	−115.88 (16)
N2—C3—C4—C5	−178.32 (17)	O11—C11—N12—C13	179.74 (18)
C3—C4—C5—C6	0.4 (3)	C10—C11—N12—C13	−2.93 (19)
C4—C5—C6—C7	−0.6 (3)	C11—N12—C13—N13	−178.18 (17)
C4—C5—C6—Br1	179.46 (14)	C11—N12—C13—N14	1.0 (2)
C5—C6—C7—C8	0.0 (3)	N13—C13—N14—C10	−179.41 (17)
Br1—C6—C7—C8	179.97 (13)	N12—C13—N14—C10	1.4 (2)
C6—C7—C8—C3	0.7 (3)	N13—C13—N14—C14	12.2 (3)
C6—C7—C8—C9	179.66 (17)	N12—C13—N14—C14	−167.02 (17)
C4—C3—C8—C7	−1.0 (3)	C11—C10—N14—C13	−2.85 (18)
N2—C3—C8—C7	177.94 (15)	C9—C10—N14—C13	115.79 (16)
C4—C3—C8—C9	179.88 (16)	C11—C10—N14—C14	165.59 (16)
N2—C3—C8—C9	−1.21 (19)	C9—C10—N14—C14	−75.8 (2)
C7—C8—C9—O9	−63.7 (3)	C1—N2—C15—C16	111.7 (2)
C3—C8—C9—O9	115.31 (17)	C3—N2—C15—C16	−76.5 (2)
C7—C8—C9—C10	63.1 (2)	N2—C15—C16—C17	−33.1 (3)
C3—C8—C9—C10	−117.84 (16)	N2—C15—C16—C21	150.87 (17)
C7—C8—C9—C1	−177.84 (18)	C21—C16—C17—C18	1.4 (3)
C3—C8—C9—C1	1.18 (18)	C15—C16—C17—C18	−174.63 (18)
O1—C1—C9—O9	54.1 (2)	C16—C17—C18—C19	−0.5 (3)
N2—C1—C9—O9	−121.83 (15)	C17—C18—C19—C20	−0.8 (3)
O1—C1—C9—C8	175.19 (17)	C18—C19—C20—C21	1.0 (3)
N2—C1—C9—C8	−0.78 (18)	C19—C20—C21—C16	0.0 (3)
O1—C1—C9—C10	−64.0 (2)	C17—C16—C21—C20	−1.2 (3)
N2—C1—C9—C10	120.05 (16)	C15—C16—C21—C20	174.94 (17)
O9—C9—C10—N14	68.89 (18)		

### Hydrogen-bond geometry (Å, º)

**Table:**

*D*—H···*A*	*D*—H	H···*A*	*D*···*A*	*D*—H···*A*
O9—H9···O1*S*^i^	0.84	1.87	2.694 (2)	169
N13—H13*A*···O11^ii^	0.81 (2)	2.00 (2)	2.811 (2)	171 (2)
N13—H13*B*···O9^iii^	0.82 (2)	2.36 (2)	2.933 (2)	128 (2)
N13—H13*B*···O1^iii^	0.82 (2)	2.46 (2)	3.158 (2)	144 (2)
O1*S*—H1*S*···N12	0.84	1.91	2.745 (2)	174

Symmetry codes: (i) *x*+1, *y*, *z*; (ii) *x*+1/2, −*y*+1/2, *z*+1/2; (iii) *x*−1/2, −*y*+1/2, *z*+1/2.

## References

[bb1] Nonius (1998). *COLLECT* Nonius BV, Delft, The Netherlands.

[bb2] Otwinowski, Z. & Minor, W. (1997). *Methods in Enzymology*, Vol. 276, *Macromolecular Crystallography*, Part A, edited by C. W. Carter Jr & R. M. Sweet, pp. 307–326. New York: Academic Press.

[bb3] Pandeya, S. N., Smitha, S., Jyoti, M. & Sridhar, S. K. (2005). *Acta Pharm.* **55**, 27–46. 15907222

[bb4] Parkin, S., Moezzi, B. & Hope, H. (1995). *J. Appl. Cryst.* **28**, 53–56.

[bb5] Penthala, N. R., Reddy, T. R. Y., Parkin, S. & Crooks, P. A. (2009*a*). *Acta Cryst.* E**65**, o552. 10.1107/S1600536809004875 PMC2968573 21582211

[bb6] Penthala, N. R., Reddy, T. R. Y., Parkin, S. & Crooks, P. A. (2009*b*). *Acta Cryst.* E**65**, o2909–o2910. 10.1107/S1600536809043797 PMC2971372 21578489

[bb7] Sheldrick, G. M. (2008). *Acta Cryst.* A**64**, 112–122. 10.1107/S0108767307043930 18156677

[bb8] Tang, Y., Chen, G., Zhang, J. & Chen, S. (2009). *Acta Cryst.* E**65**, o2597. 10.1107/S1600536809039087 PMC2971264 21578216

[bb9] Vine, K. L., Locke, J. M., Ranson, M., Benkendorff, K., Pyne, S. G. & Bremner, J. B. (2007). *Bioorg. Med. Chem.* **15**, 931–938. 10.1016/j.bmc.2006.10.035 17088067

